# Reliability of the Five Step Assessment and Its Coefficients of Impairment in Spastic Paresis

**DOI:** 10.1016/j.arrct.2025.100444

**Published:** 2025-02-22

**Authors:** Marjolaine Baude, Maud Pradines, Caroline Gault-Colas, Damien Motavasseli, David Simpson, Tymothée Poitou, Violaine Piquet, Pierre-André Natella, Jean-Michel Gracies

**Affiliations:** a Service de Rééducation Neurolocomotrice, Centre Hospitalo-Universitaire Henri Mondor, Créteil, France; b UR BIOTN, Université Paris Est Créteil (UPEC), Créteil, France; c Clinical Neurophysiology Laboratories, Neuromuscular Disorders Division, Department of Neurology, Icahn School of Medicine at Mount Sinai, New York, NY; d Unité de Recherche Clinique, Centre Hospitalo-Universitaire Henri Mondor, Créteil, France; e Service de Rééducation, Centre Hospitalier de Bastia, Université de Corse, Corte, France

**Keywords:** Five Step Assessment, Intraclass correlation coefficients, Rehabilitation, Reliability, Spasticity, Spastic paresis, Stroke

## Abstract

**Objective:**

To determine the intrarater and interrater reliability of the Five Step Assessment (FSA) and its derived coefficients of impairment in chronic spastic paresis.

**Design:**

Prospective observational study.

**Setting:**

Study of outpatients followed in a rehabilitation department.

**Participants:**

In this single-center prospective study, participants (n=18) with chronic hemiparesis (>1y since injury) were evaluated by 4 raters (3 medical doctors, 1 physiotherapist; experience in hemiparesis, 14±9y).

**Interventions:**

All raters estimated muscle shortening, spasticity, weakness, and fatigability against the resistance of 8 key antagonists in adults twice, one week apart.

**Main Outcome Measures:**

FSA involves measuring 4 angles: angle of arrest at slow speed of stretch (X_V1_), angle of catch or clonus at fast speed of stretch (X_V3_, Tardieu), angle of match between maximal agonist effort and passive and active antagonist resistances (X_A_), residual angle of match after 15 seconds of repeated maximal amplitude active efforts (X_A15_), and spasticity grade Y. Four derived coefficients of impairment were studied: coefficients of shortening, C_SH=_(X_N_−X_V1_)/X_N_ (X_N_, normally expected maximal passive joint amplitude); of spasticity, C_SP_=(X_V1_−X_V3_)/X_V1_; of weakness, C_W_=(X_V1_−X_A_)/X_V1_; and of fatigability, C_F_=(X_A_−X_A15_)/X_A_. Both intraclass correlation coefficients and mean differences were calculated for each parameter.

**Results:**

Among 18 participants (four women), intrarater reliability was good to excellent (intraclass correlation coefficient >0.75) for all parameters in all muscles. Interrater reliability was good to excellent for all muscles and parameters except for spasticity grade Y and coefficient of fatigability (moderate).

**Conclusions:**

The 5 parameters and 4 coefficients of impairment of the FSA have moderate-to-excellent intrarater and interrater reliability in chronic spastic paresis.

## Highlights

• The Five Step Assessment yields five parameters and four coefficients of impairment.
• The scale estimates the degree of muscle shortening, spasticity, weakness, and fatigability.
• The five parameters have good-to-excellent intrarater and interrater reliabilities.
• The four coefficients have moderate-to-excellent intrarater and interrater reliabilities.
• The Five Step Assessment takes a little over two minutes per muscle.

## Graphical Abstract

**Figure:**
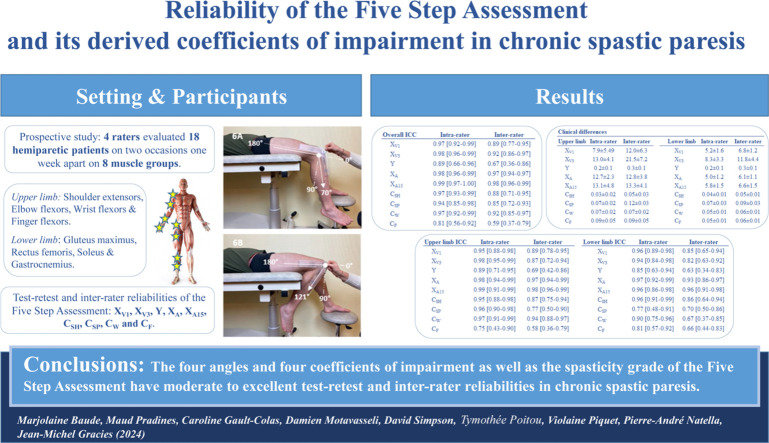

Spastic paresis results from neurological lesions involving motor command execution pathways, causing major disability and economic costs.^1^, ^2^, ^3^ Pathophysiological mechanisms include an evolving muscle disorder, *spastic myopathy*,^4^, ^5^, ^6^ and a neurological disorder impeding motor command,^1^^,^^2^ which comprises 2 components: paresis, i.e. reduced command accessing agonist motor neurons,^1^ and various forms of antagonist muscle overactivity, including *spasticity*, an exaggeration of velocity-dependent stretch reflexes detected and measured at rest^2^^,^^4^^,^^5^^,^^7^, s*pastic cocontraction*, an excessive contraction of the antagonist muscle, triggered by voluntary command directed to the agonist, sensitive to the degree of stretch imposed on the cocontracting antagonist^8^, and *spastic dystonia*, i.e. chronic tonic muscle activity at rest, sensitive to the stretch imposed on the dystonic muscle, causing esthetic prejudice and social disability.^2^^,^^4^^,^^9^^,^^10^

While research on spastic paresis has long been confined to the sole *spasticity* symptom,^11^^,^^12^ individualization of the latter forms of muscle overactivity is important. The oversimplification and lack of physiological correlates of the non–velocity- and non–range-specifying Ashworth-derived scales, such as the Modified Ashworth Scale, are today understood.^13^, ^14^, ^15^ In fact, the Clinical Outcome Assessment Program Committee of the Movement Disorders Society no longer recommends them for assessing spasticity.^16^

In that context and taking the physiological characteristics of spastic paresis into account, the Tardieu scale was created in 2000^17^^,^^18^ as an application of Tardieu's concepts for clinical examination.^19^ The first study that tested the reliability of the complete Tardieu scale, using percent agreement frequency as the statistical method of reference, was published in 2010.^20^ A number of reports have used what has been termed the “modified Tardieu scale” from our early personal communication on an unfinished version of the instrument, lacking, in particular, the definition of the spasticity angle.^21^, ^22^, ^23^

The Tardieu scale, which has recently been recommended as the most appropriate clinical tool for assessing spasticity,^16^ was later expanded into the Five Step Assessment (FSA), a stepwise quantified assessment yielding 1 functional parameter (10-meter ambulation speed for the lower limb, Modified Frenchay Scale for the upper limb) and 4 technical parameters with derived coefficients of impairment to estimate spasticity, *spastic myopathy*, and muscle resistance to single active and repeated active efforts.^5^^,^^24^ The underlying concept is that motor impairment in *chronic* spastic paresis owes more to passive and active resistance from stretched muscles than to agonist paresis itself.^1^^,^^2^^,^^4^^,^^8^ The FSA has since been used in a number of studies.^24^, ^25^, ^26^, ^27^, ^28^, ^29^, ^30^, ^31^, ^32^, ^33^, ^34^, ^35^ While intrarater and interrater reliability of the functional steps of the FSA has been reported,^34^^,^^35^ that of the technical steps of the scale is yet to be established.

The main objective of the study was to evaluate intrarater and interrater reliability of the technical parameters of the FSA and their derived coefficients of impairment in the upper and lower limbs of adults with chronic hemiparesis (over 1y after injury). Patient and rater acceptability of the assessment was also investigated.

## Methods

This study followed the Guidelines for Reporting Reliability and Agreement Studies.^36^

### Study participants

Participants were outpatients from a neurorehabilitation department, meeting the following criteria: age ≥18 and chronic spastic paresis for ≥1 year from any cause. Exclusion criteria were (1) major behavioral or cognitive impairment interfering with the ability to participate in the study; (2) severe limb pain due to skin or joint damage; (3) choreoathetosis in the paretic limbs, preventing true muscle rest; (4) cast on the paretic hemibody; (5) change in dose of systemic “antispasticity” drugs within 30 days of study; and (6) botulinum toxin injections within 6 months before enrolment.

### Raters, training, and study protocol

Three physical medicine and rehabilitation specialists and 1 physiotherapist, selected as the 4 raters of the study, evaluated resistance to movement from selected muscle groups twice 1 week apart, according to the FSA (see appendices 1-3). Four upper limb and 4 lower limb muscle groups, typically involved in functional limitations, were rated shoulder extensors, elbow, wrist, and finger flexors, and gluteus maximus, rectus femoris, soleus, and gastrocnemius.

Before the first evaluation, a written protocol (example for soleus in appendix 4) provided a standardized description of the procedure, including subject and rater positioning, passive and active stretching maneuvers, and bony landmarks to measure angles, as previously published for the lower limb.^37^ For each muscle, investigators measured 4 angles: (1) *angle of arrest* at slow speed of stretch, defined as maximal clinical muscle extensibility (X_V1_), (2) *angle of catch* or clonus at fast speed of stretch (X_V3_), with spasticity grade Y (X_V1_, X_V3_, and Y making up the Tardieu scale), (3) *angle of match* between maximal agonist effort and passive and active resistances from the antagonist (X_A_), and (4) *residual angle of match* after 15 seconds of maximal repeated alternating movements against the resistance of the tested muscle (X_A15_) (see below). During passive maneuvers (X_V1_, X_V3_), angles were estimated without a goniometer, while angles measured during active efforts (X_A_, X_A15_) were measured using manual goniometry. Four coefficients of impairment were derived from the measured angles: coefficients of shortening, C_SH_=(X_N_−X_V1_)/X_N_ (X_N_, normally expected maximal passive joint amplitude); spasticity, C_SP_=(X_V1_−X_V3_)/X_V1_; weakness, C_W_=(X_V1_−X_A_)/X_V1_; and fatigability, C_F_=(X_A_−X_A15_)/X_A_.^6^

**Fig 1:**
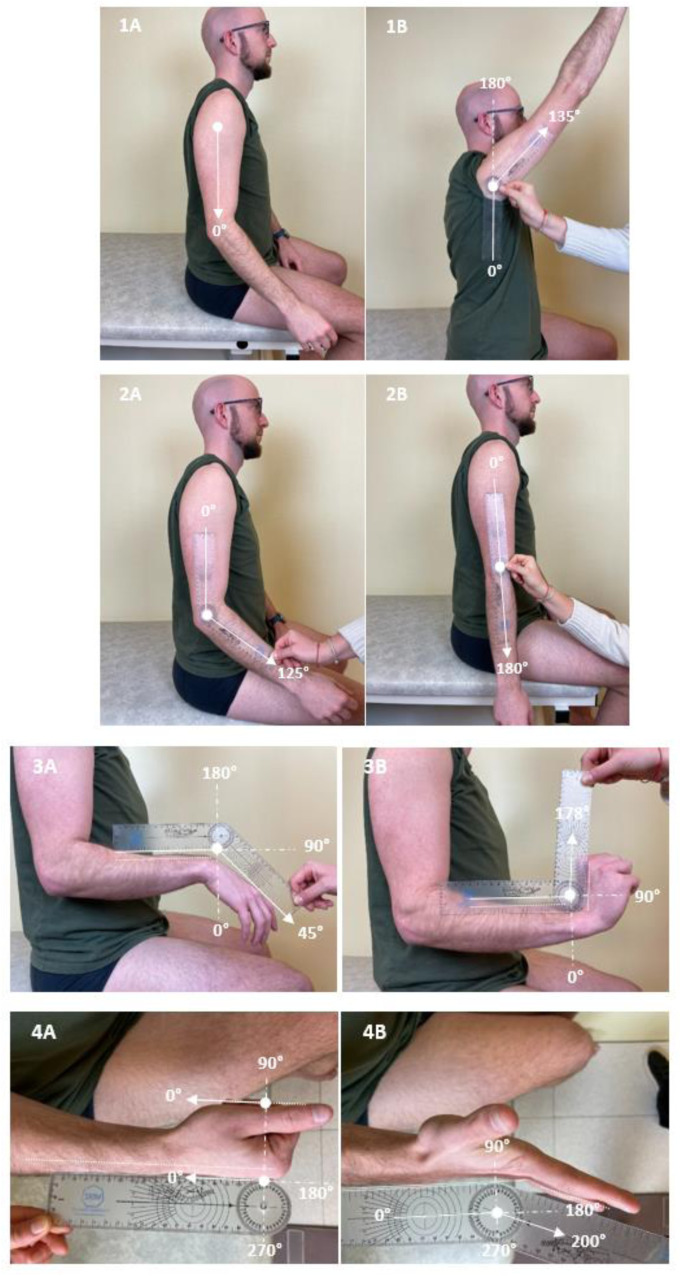

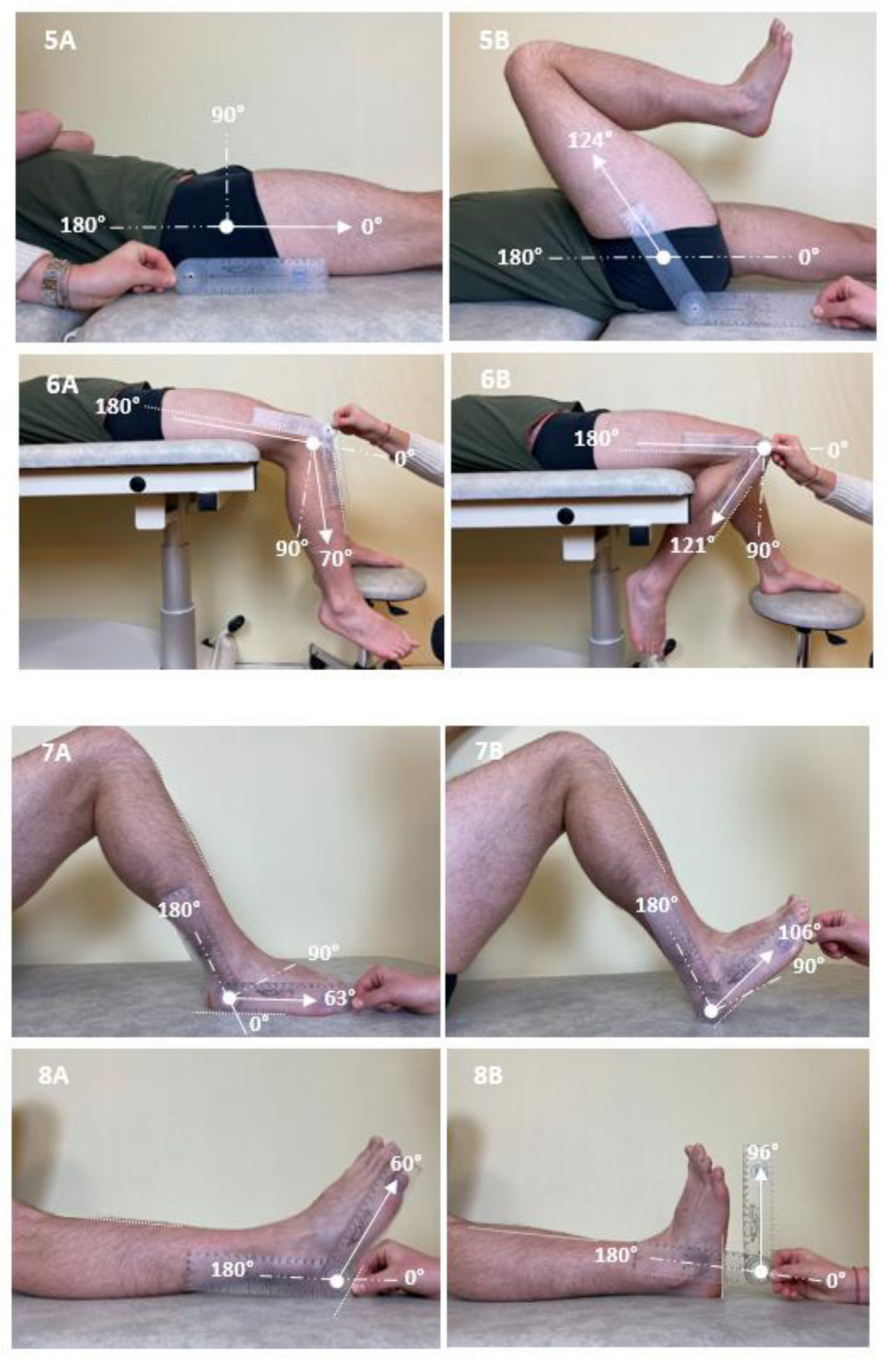
Photos illustrating measures of angles of match from rest (A) to maximal active amplitude (B) with a goniometer for the 8 testing muscle groups in one patient: shoulder extensors (1), elbow flexors (2), wrist flexors (3), finger flexors (4), gluteus maximus (5), rectus femoris (6), soleus (7), and gastrocnemius (8). Angles in (A) are shown for the purpose of clarity; only the angle in (B) is considered for the measurement of angles of match.

Figure 1 illustrates measures of the angle of match X_A_ for each muscle group evaluated. A one-hour group training session was organized before the study evaluations, in which the group of raters evaluated the 8 muscles in a subject who was not involved in the reliability study. The protocol was reviewed, and raters received feedback on their practice.^20^

During study evaluations, each rater reported data on individual observation sheets. Any complaints or comments from participants were noted. Each rater was blinded to the other raters’ data. Time to run each assessment was recorded. Each participant rested for 30 minutes between 2 consecutive assessments.

### Procedures involved in the FSA

The evaluation comprises 5 consecutive steps (see appendices 1-4). Step 1 evaluates active function using the Modified Frenchay Scale for the upper limb and the 10-meter Ambulation Test for the lower limb; step 1 was not investigated here as the reliability of these assessments has been previously demonstrated.^34^^,^^35^ Steps 2-5 are described in appendix 1, evaluation grids are proposed in appendices 2 and 3, and the procedure is illustrated for the soleus muscle in appendix 4.

### Bias limitation

(1) Assessors were specialized in spastic paresis and familiar with the scale studied; (2) patients were in chronic stages of hemiparesis; (3) group training was organized prior to study start; and (4) a detailed written assessment protocol was available for each muscle (an example for the soleus muscle is in appendix 4).

### Study size

Given the large number of muscles (n=8 per subject) and parameters (n=5 per muscle), as well as the number of evaluators (n=4), the number of subjects required for this reliability study was set at 20. Indeed, the total number of FSAs carried out would be 80 each week, 160 in total; the number of parameters measured would be thus 800 (five parameters directly measured: X_V1_, X_V3_, Y, X_A_, X_A15_). In a recent literature review that identified 33 investigations of the reliability of Modified Ashworth Scale, the mean number of participants was 20, and the mean number of muscles studied was 4.^38^

### Statistical analysis

Quantitative variables were described using means or medians depending on normality. Qualitative variables were presented using n (%). For quantitative parameters (X_V1_, X_V3_, X_A_, X_A15_, C_SH_, C_SP_, C_W_, C_F_), we measured intrarater (stricto sensu test–retest) reliability using intrarater intraclass correlation coefficients (ICCs) for the measures performed 1 week apart^39^ and interrater reliability using ICCs for angles measured by the 4 raters on the same day on each participant; the mean interrater reliability over the 2 study visits was reported. A two-way random, absolute agreement ICC model was chosen on the assumption that results could be generalized to any similar raters. Based on the 95% confidence intervals of ICC estimates, agreement was interpreted as excellent if >0.90, good if between 0.75 and 0.90, moderate if between 0.50 and 0.75, and poor if <0.50.^40^ For Y (ordinal data), we calculated Fleiss’ κ and Gwet's agreement coefficient (AC).^41^^,^^42^ Agreement was considered close to perfect for κ between 0.81 and 0.99, strong between 0.61 and 0.80, moderate between 0.41 and 0.60, weak between 0.21 and 0.40, and poor <0.21.^43^^,^^44^ We also calculated the mean intrarater and interrater differences between Y and joint angle measurements, as well as their percentage of the maximal physiologic amplitudes X_N_ defined as follows: shoulder extensors, 180°; elbow flexors, 180°; wrist flexors, 180°; finger flexors, 270°; gluteus maximus, 150°, rectus femoris, 150°; soleus, 120; and gastrocnemius, 115°.^32^ For missing data, imputations by mean or median were used. All tests used Stata software 15.0 (Release 15; StataCorp LP).

### Ethics

The study protocol was approved by the local Institutional Review Board on March 25, 2021 (institutional review board number 00011558; advice number 2021-114). All participants were provided with the study information note and signed their nonopposition to anonymous use of their data.

## Results

### Participants

#### Subjects

Eighteen subjects with chronic spastic paresis (age, 50±14y; 78% men) were recruited (tables 1 and 2). Mean time since injury was 5.3±2.4 years. Sixteen had hemiparesis (right, 63%), while 2 had bilateral hemiparesis (one double stroke, one cervical spinal cord injury). Causes were vascular (n=16), traumatic (spinal cord injury; n=1), and infectious (treated abscess; n=1).

**Table 1:** Characteristics of participants

Raters	
Number	4
Age (y)	47±11
Women	3 (75)
Experience with spastic paresis (y)	14±9
Experience with FSA (y)	8±7
PM&R physicians	3 (75)
Physical therapists	1 (25)
Participants	
Number	18
Age (y)	50±14
Women	3 (21)
Side of hemiparesis	
Right	4 (21)
Left	12 (63)
Bilateral	2 (16)
Right-handed	18 (100)
Time since injury (y)	5.3±2.4
Cause	
Ischemic stroke	10 (56)
Hemorrhagic stroke	6 (33)
Other (SCI, cerebral abscess)	2 (11)

NOTE. All data are mean ± SD for quantitative data and n (%) for qualitative measures. Abbreviations: FSA, Five Step Assessment; PM&R, physical medicine and rehabilitation; SCI, spinal cord injury.

**Table 2:** Mean (all raters) FSA parameters at visit 1 per muscle group

Shoulder extensors				Gluteus maximus			
X_V1_	142.8±26.2	C_SH_	0.21±0.14	X_V1_	126.7±17.8	C_SH_	0.16±0.12
X_V3_	108.2±38.2	C_SP_	0.25±0.18	X_V3_	110.5±22.4	C_SP_	0.13±0.09
Y	1.8±0.4			Y	1.7±0.4		
X_A_	111.1±37.9	C_W_	0.24±0.18	X_A_	114.2±14.9	C_W_	0.10±0.07
X_A15_	97.6±41.7	C_F_	0.14±0.12	X_A15_	107.6±16.3	C_F_	0.06±0.05
Elbow flexors				Rectus femoris			
X_V1_	173.9±15.9	C_SH_	0.04±0.09	X_V1_	129.3±15.8	C_SH_	0.14±0.10
X_V3_	127.3±33.3	C_SP_	0.27±0.17	X_V3_	72.4±37.9	C_SP_	0.45±0.25
Y	1.9±0.4			Y	2.0±0.6		
X_A_	158.9±26.6	C_W_	0.09±0.11	X_A_	100.7±13.9	C_W_	0.22±0.10
X_A15_	148.4±28.6	C_F_	0.07±0.06	X_A15_	88.6±17.6	C_F_	0.12±0.12
Wrist flexors				Soleus			
X_V1_	169.6±15.9	C_SH_	0.07±0.07	X_V1_	102.1±9.3	C_SH_	0.15±0.07
X_V3_	129.5±37.8	C_SP_	0.25±0.17	X_V3_	91.6±7.7	C_SP_	0.10±0.07
Y	2.0±0.5			Y	2.3±0.6		
X_A_	135.4±31.2	C_W_	0.20±0.17	X_A_	93.2±10.7	C_W_	0.09±0.07
X_A15_	129.3±30.9	C_F_	0.08±0.11	X_A15_	88.6±10.9	C_F_	0.05±0.05
Finger flexors				Gastrocnemius			
X_V1_	251.0±57.4	C_SH_	0.14±0.18	X_V1_	94.6±8.5	C_SH_	0.18±0.07
X_V3_	171.6±70.1	C_SP_	0.32±0.21	X_V3_	82.6±8.4	C_SP_	0.12±0.07
Y	2.1±0.6			Y	2.2±0.5		
X_A_	152.9±91.5	C_W_	0.41±0.30	X_A_	80.3±12.6	C_W_	0.16±0.10
X_A15_	132.4±92.3	C_F_	0.19±0.11	X_A15_	76.1±12.8	C_F_	0.06±0.06

NOTE. All data are quantitative and displayed as mean ± SD.

Abbreviations: C_F_, coefficient of fatigability; C_SH_, coefficient of shortening; C_SP_, coefficient of spasticity; C_W_, coefficient of weakness; X_A_, angle of match between maximal agonist efforts and passive and active resistances from the tested muscle (maximal active range of motion); X_A15_, residual angle of match after 15 seconds of repeated maximal amplitude active movements against the resistance of the tested muscle; X_V1_, maximal clinical extensibility; X_V3_, angle of catch or clonus; Y, grade of spasticity.

#### Raters

The 4 raters (3 women; 3 medical doctors and 1 physiotherapist) had experience in the assessment of hemiparesis of 14±9 years. Two had extensive experience using the FSA (8±7y) in clinical practice or international multicentric trials.^28^^,^^45^ The other 2 were less experienced in using the scale (1.0±0.7y).

#### FSA evaluations at baseline

The mean parameters measured at baseline are presented in table 2 for each muscle group. The most shortened muscle groups (C_SH_>10%) were the lower limb muscles, shoulder extensors, and finger flexors; as expected, every muscle was significantly spastic (C_SP_>10%), especially rectus femoris, finger flexors, elbow flexors, shoulder extensors, and wrist flexors (C_SP_>20%); active movement was most impaired (C_W_>10%) against the resistance of finger flexors, shoulder extensors, rectus femoris, wrist flexors, and gastrocnemius and most fatigable (C_F_>10%) against finger flexors, shoulder extensors, and rectus femoris.

### Intrarater reliability (stricto sensu test–retest)

#### ICCs

Across all muscles (table 3), intrarater ICCs were good to excellent (ICC>0.75) for all parameters, ranging from 0.81 (0.56-0.92) to 0.99 (0.96-0.99). When considering individual muscle data, intrarater ICCs were also moderate to excellent (ICC>0.50) for all parameters, except for C_F_ in soleus, where it was poor. For individual coefficients of impairment, intrarater ICCs were as follows: excellent for C_SH_ (ICC>0.90) in all muscles except for rectus femoris (good); excellent for C_SP_ in upper limb muscles and good to excellent for C_SP_ in lower limb muscles; excellent for C_W_ in upper limb muscles and plantar flexors, and good in gluteus maximus and moderate (0.50<ICC<0.75) in rectus femoris; moderate to good for C_F_ in all muscles except soleus (<0.50).

**Table 3:** Intrarater and interrater ICCs (95% CI) per muscle and per parameter

	Intrarater ICC (95% CI)	Interrater ICC (95% CI)		Intrarater ICC (95% CI)	Interrater ICC (95% CI)
Shoulder extensors			Gluteus maximus		
X_V1_	0.96 (0.91-0.99)	0.90 (0.76-0.96)	X_V1_	0.93 (0.93-0.99)	0.93 (0.86-0.97)
X_V3_	0.98 (0.95-0.99)	0.81 (0.60-0.92)	X_V3_	0.93 (0.83-0.97)	0.88 (0.78-0.95)
Y	0.93 (0.82-0.97)	0.58 (0.35-0.78)	Y	0.52 (0.08-0.79)	0.41 (0.16-0.67)
X_A_	0.99 (0.98-1.00)	0.97 (0.93-0.99)	X_A_	0.96 (0.89-0.98)	0.85 (0.61-0.94)
X_A15_	0.98 (0.96-0.99)	0.96 (0.92-0.98)	X_A15_	0.96 (0.87-0.98)	0.91 (0.81-0.96)
C_SH_	0.96 (0.90-0.99)	0.90 (0.78-0.96)	C_SH_	0.97 (0.93-0.99)	0.93 (0.87-0.97)
C_SP_	0.93 (0.82-0.98)	0.48 (0.20-0.73)	C_SP_	0.62 (0.23-0.83)	0.55 (0.32-0.78)
C_W_	0.98 (0.95-0.99)	0.91 (0.83-0.96)	C_W_	0.77 (0.33-0.92)	0.41 (0.17-0.67)
C_F_	0.79 (0.53-0.92)	0.57 (0.35-0.78)	C_F_	0.73 (0.40-0.89)	0.36 (0.12-0.63)
Elbow flexors			Rectus femoris		
X_V1_	0.99 (0.96-0.99)	0.96 (0.92-0.98)	X_V1_	0.78 (0.50-0.91)	0.67 (0.46-0.84)
X_V3_	0.97 (0.91-0.99)	0.86 (0.74-0.94)	X_V3_	0.91 (0.77-0.96)	0.70 (0.48-0.86)
Y	0.84 (0.62-0.93)	0.78 (0.63-0.90)	Y	0.82 (0.58-0.93)	0.47 (0.24-0.71)
X_A_	0.97 (0.91-0.99)	0.92 (0.85-0.97)	X_A_	0.92 (0.80-0.97)	0.81 (0.66-0.92)
X_A15_	0.97 (0.92-0.99)	0.92 (0.86-0.97)	X_A15_	0.90 (0.74-0.96)	0.78 (0.62-0.90)
C_SH_	0.98 (0.96-0.99)	0.96 (0.93-0.98)	C_SH_	0.76 (0.47-0.90)	0.69 (0.50-0.85)
C_SP_	0.95 (0.88-0.98)	0.82 (0.68-0.92)	C_SP_	0.85 (0.64-0.94)	0.71 (0.50-0.86)
C_W_	0.91 (0.79-0.97)	0.84 (0.70-0.93)	C_W_	0.67 (0.29-0.86)	0.46 (0.23-0.71)
C_F_	0.69 (0.33-0.87)	0.38 (0.15-0.65)	C_F_	0.64 (0.25-0.85)	0.54 (0.32-0.76)
Wrist flexors			Soleus		
X_V1_	0.94 (0.84-0.98)	0.61 (0.39-0.81)	X_V1_	0.97 (0.91-0.99)	0.73 (0.39-0.89)
X_V3_	0.97 (0.91-0.99)	0.77 (0.55-0.90)	X_V3_	0.81 (0.57-0.92)	0.34 (0.05-0.64)
Y	0.67 (0.32-0.86)	0.51 (0.27-0.74)	Y	0.91 (0.75-0.97)	0.83 (0.69-0.92)
X_A_	0.94 (0.85-0.98)	0.90 (0.81-0.96)	X_A_	0.99 (0.96-0.99)	0.89 (0.81-0.96)
X_A15_	0.94 (0.84-0.98)	0.89 (0.79-0.95)	X_A15_	0.96 (0.89-0.99)	0.92 (0.86-0.97)
C_SH_	0.93 (0.82-0.97)	0.59 (0.34-0.80)	C_SH_	0.96 (0.90-0.98)	0.73 (0.38-0.89)
C_SP_	0.95 (0.88-0.98)	0.78 (0.58-0.90)	C_SP_	0.80 (0.54-0.92)	0.39 (0.14-0.66)
C_W_	0.92 (0.81-0.97)	0.82 (0.68-0.92)	C_W_	0.91 (0.76-0.97)	0.51 (0.25-0.74)
C_F_	0.55 (0.14-0.80)	0.30 (0.08-0.57)	C_F_	0.33 (0.00-0.68)	0.13 (0.00-0.41)
Finger flexors			Gastrocnemius		
X_V1_	0.92 (0.79-0.97)	0.81 (0.65-0.92)	X_V1_	0.94 (0.84-0.98)	0.73 (0.41-0.88)
X_V3_	0.97 (0.93-0.99)	0.84 (0.70-0.93)	X_V3_	0.92 (0.80-0.97)	0.52 (0.23-0.76)
Y	0.89 (0.73-0.96)	0.58 (0.33-0.79)	Y	0.77 (0.48-0.91)	0.69 (0.50-0.85)
X_A_	0.96 (0.87-0.98)	0.94 (0.89-0.98)	X_A_	0.96 (0.89-0.98)	0.91 (0.83-0.96)
X_A15_	0.96 (0.39-0.99)	0.96 (0.92-0.98)	X_A15_	0.98 (0.95-0.99)	0.93 (0.86-0.97)
C_SH_	0.89 (0.74-0.96)	0.76 (0.59-0.89)	C_SH_	0.94 (0.58-0.98)	0.72 (0.41-0.88)
C_SP_	0.90 (0.76-0.96)	0.61 (0.36-0.81)	C_SP_	0.71 (0.37-0.88)	0.35 (0.13-0.62)
C_W_	0.91 (0.69-0.97)	0.91 (0.83-0.96)	C_W_	0.93 (0.81-0.97)	0.73 (0.42-0.89)
C_F_	0.63 (0.14-0.86)	0.56 (0.34-0.77)	C_F_	0.82 (0.57-0.93)	0.64 (0.43-0.82)
Upper limb			Lower limb		
X_V1_	0.95 (0.88-0.98)	0.89 (0.78-0.95)	X_V1_	0.96 (0.89-0.98)	0.85 (0.65-0.94)
X_V3_	0.98 (0.95-0.99)	0.87 (0.72-0.94)	X_V3_	0.94 (0.84-0.98)	0.82 (0.63-0.92)
Y	0.89 (0.71-0.95)	0.69 (0.42-0.86)	Y	0.85 (0.63-0.94)	0.63 (0.34-0.83)
X_A_	0.98 (0.94-0.99)	0.97 (0.94-0.99)	X_A_	0.97 (0.92-0.99)	0.93 (0.86-0.97)
X_A15_	0.99 (0.91-0.99)	0.98 (0.96-0.99)	X_A15_	0.96 (0.86-0.98)	0.96 (0.91-0.98)
C_SH_	0.95 (0.88-0.98)	0.87 (0.75-0.94)	C_SH_	0.96 (0.91-0.99)	0.86 (0.64-0.94)
C_SP_	0.96 (0.90-0.98)	0.77 (0.50-0.90)	C_SP_	0.77 (0.48-0.91)	0.70 (0.50-0.86)
C_W_	0.97 (0.91-0.99)	0.94 (0.88-0.97)	C_W_	0.90 (0.75-0.96)	0.67 (0.37-0.85)
C_F_	0.75 (0.43-0.90)	0.58 (0.36-0.79)	C_F_	0.81 (0.57-0.92)	0.66 (0.44-0.83)
Overall	Intrarater	Interrater			

X_V1_	0.97 (0.92-0.99)	0.89 (0.77-0.95)			
X_V3_	0.98 (0.96-0.99)	0.92 (0.86-0.97)			
Y	0.89 (0.66-0.96)	0.67 (0.36-0.86)			
X_A_	0.98 (0.96-0.99)	0.97 (0.94-0.97)			
X_A15_	0.99 (0.97-1.00)	0.98 (0.96-0.99)			
C_SH_	0.97 (0.93-0.99)	0.88 (0.71-0.95)			
C_SP_	0.94 (0.85-0.98)	0.85 (0.72-0.93)			
C_W_	0.97 (0.92-0.99)	0.92 (0.85-0.97)			
C_F_	0.81 (0.56-0.92)	0.59 (0.37-0.79)			

Abbreviations: C_F_, coefficient of fatigability; CI, confidence interval; C_SH_, coefficient of shortening; C_SP_, coefficient of spasticity; C_W_, coefficient of weakness; ICC, intraclass correlation coefficient; X_A_, angle of match between agonist effort and passive and active resistances from the antagonist, that is, maximal active range of motion against the resistance of the tested muscle; X_A15_, residual angle of match after 15 seconds of repeated maximal amplitude active movements against the resistance of the tested muscle; X_V1_, maximal clinical extensibility; X_V3_, angle of catch or clonus; Y, grade of spasticity.

#### Mean intrarater differences

For the raw angle measurements (X_V1_, X_V3_, X_A_, X_A15_), the mean intrarater differences represented <5% of X_N_ for shoulder extensors, elbow flexors, and soleus and <10% for all other muscle groups (table 4). For the derived coefficients of impairment, the mean intrarater differences were <5% for plantar flexors and <10% for all other muscles except for rectus femoris C_SP_ (12%) and finger flexor C_W_ (12%) and C_F_ (17%) (table 4). Intrarater Fleiss’ κ coefficients for Y were almost perfect for soleus, strong for elbow flexors, finger flexors, rectus femoris, and gastrocnemius, moderate for shoulder extensors and wrist flexors, and weak for gluteus maximus (supplemental tables S1 and S2, available online only at http://www.archives-pmr.org/).

**Table 4:** Mean intrarater and interrater differences per parameter, per muscle group, and for overall limb

Muscles	Intrarater	Interrater		Intrarater	Interrater
Shoulder extensors			Gluteus maximus		
X_V1_	8.3±5.9 (0.04)	11.2±4.6 (0.06)	X_V1_	6.2±3.3 (0.04)	7.0±2.3 (0.05)
X_V3_	8.5±3.7 (0.05)	18.6±10.4 (0.10)	X_V3_	9.5±5.5 (0.06)	10.0±5.1 (0.07)
Y	0.1±0.1 (0.02)	0.2±0.2 (0.05)	Y	0.2±0.3 (0.05)	0.3±0.3 (0.07)
X_A_	6.9±2.4 (0.04)	8.9±2.7 (0.05)	X_A_	5.6±2.5 (0.04)	7.4±2.5 (0.05)
X_A15_	8.1±6.3 (0.04)	11.3±4.3 (0.06)	X_A15_	6.3±2.7 (0.04)	6.8±2.5 (0.04)
C_SH_	0.04±0.03	0.06±0.03	C_SH_	0.04±0.02	0.04±0.02
C_SP_	0.07±0.04	0.15±0.07	C_SP_	0.07±0.04	0.07±0.03
C_W_	0.06±0.03	0.07±0.02	C_W_	0.05±0.02	0.06±0.02
C_F_	0.08±0.06	0.10±0.06	C_F_	0.04±0.02	0.05±0.02
Elbow flexors			Rectus femoris		
X_V1_	2.5±2.5 (0.01)	3.4±2.7 (0.02)	X_V1_	7.8±5.1 (0.04)	9.2±3.3 (0.06)
X_V3_	9.9±5.9 (0.05)	13.0±8.6 (0.07)	X_V3_	15.8±9.6 (0.10)	22.8±15.3 (0.15)
Y	0.2±0.2 (0.05)	0.1±0.2 (0.03)	Y	0.2±0.2 (0.05)	0.5±0.2 (0.12)
X_A_	5.6±7.1 (0.03)	6.5±6.5 (0.04)	X_A_	5.7±2.4 (0.04)	7.3±2.9 (0.05)
X_A15_	8.3±6.3 (0.05)	8.9±7.2 (0.05)	X_A15_	8.4±4.5 (0.06)	10.4±3.7 (0.07)
C_SH_	0.01±0.01	0.02±0.01	C_SH_	0.05±0.03	0.05±0.02
C_SP_	0.06±0.03	0.08±0.05	C_SP_	0.12±0.09	0.16±0.09
C_W_	0.04±0.04	0.04±0.04	C_W_	0.06±0.03	0.08±0.02
C_F_	0.05±0.02	0.05±0.02	C_F_	0.08±0.05	0.08±0.04
Wrist flexors			Soleus		
X_V1_	5.8±3.6 (0.03)	11.2±6.2 (0.06)	X_V1_	3.3±1.3 (0.03)	6.0±1.8 (0.05)
X_V3_	12.2±5.5 (0.07)	20.5±12.0 (0.11)	X_V3_	4.0±1.9 (0.03)	7.6±2.6 (0.06)
Y	0.3±0.2 (0.07)	0.3±0.2 (0.07)	Y	0.1±0.2 (0.02)	0.2±0.2 (0.05)
X_A_	10.6±8.1 (0.06)	12.0±6.5 (0.07)	X_A_	3.6±1.4 (0.03)	4.4±1.7 (0.04)
X_A15_	11.9±6.7 (0.07)	12.7±7.4 (0.07)	X_A15_	4.3±1.8 (0.03)	4.4±1.4 (0.04)
C_SH_	0.03±0.02	0.05±0.03	C_SH_	0.03±0.01	0.05±0.02
C_SP_	0.07±0.03	0.10±0.04	C_SP_	0.03±0.02	0.06±0.03
C_W_	0.07±0.04	0.08±0.04	C_W_	0.04±0.02	0.06±0.02
C_F_	0.06±0.05	0.07±0.05	C_F_	0.04±0.02	0.05±0.02
Finger flexors			Gastrocnemius		
X_V1_	14.9±16.1 (0.05)	22.1±21.3 (0.08)	X_V1_	3.3±1.8 (0.03)	5.2±2.5 (0.04)
X_V3_	21.4±9.5 (0.08)	34.0±14.0 (0.12)	X_V3_	3.7±1.9 (0.03)	6.6±2.1 (0.06)
Y	0.2±0.3 (0.05)	0.3±0.2 (0.07)	Y	0.2±0.3 (0.05)	0.2±0.3 (0.05)
X_A_	27.9±10.7 (0.10)	23.9±15.3 (0.09)	X_A_	5.2±2.1 (0.04)	5.3±1.6 (0.05)
X_A15_	24.0±12.7 (0.09)	20.3±13.3 (0.07)	X_A15_	4.2±1.7 (0.04)	5.1±1.8 (0.04)
C_SH_	0.04±0.06	0.07±0.08	C_SH_	0.03±0.01	0.04±0.02
C_SP_	0.10±0.04	0.15±0.06	C_SP_	0.04±0.02	0.06±0.03
C_W_	0.12±0.07	0.09±0.05	C_W_	0.05±0.02	0.07±0.02
Overall upper limb			Overall lower limb		
X_V1_	7.9±5.49	12.0±6.3	X_V1_	5.2±1.6	6.8±1.2
X_V3_	13.0±4.1	21.5±7.2	X_V3_	8.3±3.3	11.8±4.4
Y	0.2±0.1	0.3±0.1	Y	0.2±0.1	0.3±0.1
X_A_	12.7±2.3	12.8±3.8	X_A_	5.0±1.2	6.1±1.1
X_A15_	13.1±4.8	13.3±4.1	X_A15_	5.8±1.5	6.6±1.5
C_SH_	0.03±0.02	0.05±0.03	C_SH_	0.04±0.01	0.05±0.01
C_SP_	0.07±0.02	0.12±0.03	C_SP_	0.07±0.03	0.09±0.03
C_W_	0.07±0.02	0.07±0.02	C_W_	0.05±0.01	0.06±0.01
C_F_	0.09±0.05	0.09±0.05	C_F_	0.05±0.01	0.06±0.01

NOTE. All results are mean ± SD. In brackets is the percentage with respect to the maximum expected amplitude X_N_.

Abbreviations: C_F_, coefficient of fatigability; C_SH_, coefficient of shortening; C_SP_, coefficient of spasticity; C_W_, coefficient of weakness; X_A_, angle of match between agonist effort and passive and active resistances from the antagonist, that is, maximal active range of motion against the resistance of the tested muscle; X_A15_, residual angle of match after 15 seconds of repeated maximal amplitude active movements against the resistance of the tested muscle; X_V1_, maximal clinical extensibility; X_V3_, angle of catch or clonus; Y, grade of spasticity.

### Interrater reliability

#### ICCs

Across all muscles (table 3), interrater ICCs were good to excellent for all parameters except for C_F,_ which was moderate (ICC=0.59). When considering individual muscle data, for raw angle measurements, interrater ICCs were moderate to excellent in all muscles except for soleus X_V3_. For the derived coefficients, interrater ICCs were moderate to excellent for C_SH_ (ICC>0.50), moderate to good for C_SP_ except for shoulder extensors and plantar flexors (low ICC<0.50), moderate to excellent for C_W_ except for gluteus maximus and rectus femoris (low ICC<0.50), and low to moderate C_F_ (ICC<0.75).

#### Mean interrater differences

For raw angle measurements, mean interrater differences (table 4) represented <10% of X_N_ except for X_V3_ of wrist and finger flexors and rectus femoris, where they were ≤15% of X_N_. For the derived coefficients, the mean interrater differences represented <10% except for shoulder extensors, finger flexors, and rectus femoris C_SP_ (15%-16%) and for finger flexor C_F_ (17%; table 4). Interrater Fleiss’ κ coefficients for Y were >0.40 (moderate to almost perfect agreement) except for gluteus maximus with fair agreement (supplemental tables S1 and S2).

### Time consumption and tolerance

The mean time to assess the 8 muscle groups was 18±6 minutes, that is, a little over 2 minutes per muscle, which was deemed acceptable for both subjects and raters. Tolerance of evaluations was good for both subjects and raters.

## Discussion

This reliability study of the FSA (technical parameters X_V1_, X_V3_, X_A_, X_A15_, C_SH_, C_SP_, C_W_, C_F_, Y) demonstrated good-to-excellent intrarater (stricto sensu test–retest) reliability across 8 key muscle groups in chronic spastic paresis. Interrater reliability was also good to excellent across all muscles for all parameters, except for C_F_ (moderate reliability).

### Clinical relevance of a quantitative tool to evaluate the various components of spastic paresis at the bedside

Most therapeutic trials in the field have used Ashworth-derived scores as primary outcomes, including the Modified Ashworth scale as primary outcomes, with the assumption that these tools would measure *spasticity*. However, they actually assess resistance to passive movement of any origin, including muscle shortening.^13^, ^14^, ^15^, ^16^, ^17^, ^18^ Yet, these instruments became a de facto criterion standard even though conceptual or methodological validation did not occur.^13^, ^14^, ^15^ The FSA is an expansion of the Tardieu scale, which was created and named in 2000 from Tardieu's original clinical method.^17^^,^^18^ A clinical tool aiming to estimate the various roles played by spastic myopathy (X_V1_, C_SH_), spasticity (X_V3_, C_SP_), the combination of spastic cocontraction together with weakness of the agonist command (X_A_, C_W_), and fatigability of motor command (X_A15_, C_F_) may be of practical and theoretical importance.

This stepwise clinical assessment of the power of nuisance of each antagonist muscle aims to help guide therapeutic indications. When step 2 (X_V1_, C_SH_) suggests significant loss of clinical extensibility (eg, C_SH_>10%), this may drive the clinician to use lengthening interventions (eg, stretch programs) together with blocking injections on the evaluated antagonist. If step 4 (X_A_, C_W_) indicates major weakness of command (eg, C_W_>15%), or step 5 (X_A15_, C_F_) shows high levels of fatigability despite minor shortening at step 2, this may bring the clinician to focus treatment on training the motor command (eg, nonassisted alternating movement programs) along with cautious blocking injections on the evaluated muscle group. As for step 3 (X_V3_, C_SP_), this may serve, in particular, as a highly responsive indicator of how well a muscle was blocked by a focal injection.^33^^,^^46^

### Reliability statistics

What we have called *intrarater* reliability in this study was, in fact, *test–retest* reliability, as the former corresponds to the agreement between repeated observations of the same test, that is, using a videotape.^47^ We used various statistical tools to measure both intrarater and interrater reliability—ICC, mean differences, Fleiss’ κ, and Gwet's AC—to enhance robustness of the findings. Most studies in neurorehabilitation have only used ICCs for quantitative variables and κ coefficients for ordinal variables.^48^, ^49^, ^50^ In the present investigation on the reliability of Y, we found a high percentage of agreement among the raters even though Fleiss’ κ was relatively low. This phenomenon, known as the “paradox of κ,” is due to raters rarely selecting some of the available possibilities.^51^^,^^52^ In the present study, the paradox occurred because grades 1 and 4 are less frequently rated than grades 2 or 3. Therefore, the use of Gwet's AC is better adapted in this case (see supplemental tables S1 and S2).

As for joint angle measurements, we have displayed the mean intrarater and interrater differences for the sake of relevance to clinical practice. In table 4, which reports the mean intrarater and interrater rater differences between ratings, some differences may seem large at first but turn out to be small when referring to the expected maximal passive amplitude X_N_. Of note, among the studies on the reliability of the “modified Tardieu scale,”^21^, ^22^, ^23^ the study by Li et al^23^ supports the external validity of the present findings, with lower ICCs for X_V3_.

### Study limitations

The present study assessed reliability of the FSA, not its validity. Correlations with function of some technical parameters, particularly X_V1_ in the lower limb and X_A_ in the upper limb, have been demonstrated elsewhere.^25^^,^^31^^,^^53^ Additional studies will be required to determine how these parameters compare to actual three-dimensional or other types of instrumented amplitude measurements. In addition, the small sample size makes extrapolation to other work settings difficult; international multicentric studies would enhance robustness.

In the present study, the cause of spastic paresis was mostly vascular, which might also preclude conclusions about other causes. We may indeed hypothesize that some components of spastic paresis predominate depending on the condition: for example, people with multiple sclerosis might have less shortened muscles but more fatigable command, while people with cerebral palsy would be characterized by more shortened muscles.^54^ If such hypotheses are confirmed, the FSA might allow to determine and focus treatment depending on the predominant component of spastic paresis.

## Conclusions

The FSA is practical to use at the clinic or at the bedside for both patients and raters (lightly over two 2minutes per muscle), with good-to-excellent intrarater reliability and moderate-to-excellent interrater reliability for both angle and grade parameters in adults with chronic spastic paresis.

## Disclosure

M.B., C.G.-C., D.M., and J.-M.G. have some financial disclosures with Merz, Ipsen, and Allergan. M.P. has some financial disclosures with Ipsen, Merz, and Wandercraft. V.P. has some financial disclosures with Allergan and Merz. These disclosures are not related to this study. The other authors have nothing to disclose.

## Acknowledgments

We are grateful to the participants who helped us carry out the study. We also thank Emma Tison, Bryan Baguet, and Lionel Friederich for their help in producing the photographs.

## Data statements

Raw data associated with the paper are available from the corresponding author upon reasonable request.

## Appendices

### Appendix 1 Procedures involved in the Five Step Assessment

Following and expanding on Tardieu's insights on *chronic* spastic paresis, and in contrast with the assessment in peripheral paresis (Medical Research Council scale) that assesses *agonist* muscles for their capacity to generate movement against gravity, the 4 technical steps (steps 2-5) of the Five Step Assessment evaluate each muscle group for its capacity to *oppose* movement, not to generate it. Steps 2 and 3 rate capacity to oppose *passive* movements at 2 different velocities, while steps 4 and 5 rate the capacity to oppose active attempts at movement, before and after fatigue.

Each step of the assessment yields a quantitative continuous variable, making it possible to monitor status. For the technical steps, the angles determined are not based on joint positions but based on 0 being the theoretical angle of minimal stretch of the muscle group assessed. This point of minimal stretch of the muscle group is taken as the point of reference where resistance starts. Steps 2 and 3 constitute what has been known in the literature as the Tardieu scale. These 2 angles have yielded good-to-excellent intrarater and interrater reliability, both in children and adults, without the use of a goniometer.

### Step-2: angle of arrest at slow speed of stretch

Each muscle group is first evaluated using very slow and powerful stretch, without jeopardizing soft tissue integrity or patient tolerance. The angle at which muscle resistance is no longer overcome by the examiner with respect to soft tissue integrity and patient comfort is defined as the maximal passive clinical extensibility of the muscle group assessed. The angle of arrest at slow speed of stretch (X_V1_) *mostly* reflects muscle shortening (as opposed to joint adherences), more so than muscle overactivity (spastic dystonia), as it undergoes little change after lidocaine blocks or repeated botulinum toxin injections.

X_V1_ is then appreciated with respect to the expected normal passive amplitude, X_N_, defining the ratio (X_N_–X_V1_)/X_N_ as the coefficient of shortening of the tested muscle.

### Step-3: angle of catch or clonus and spasticity grade—the Tardieu scale

Each muscle group is then evaluated using fast stretch, that is, stretch at the fastest possible speed for the examiner (V3, fast velocity), *without forcing the way beyond the muscle catch*. According to the Tardieu scale, the clinician derives 2 parameters from this maneuver. The angle of catch or clonus (X_V3_) represents the threshold to elicit the reflex. The *spasticity grade* (Y), that is, the type of muscle reaction that occurs upon fast stretch at that angle, clinically reflects the *gain* of the stretch reflex. Five situations may occur as follows: (1) no muscle contraction upon fast stretch: Y=0 (X_V1_=X_V3_); (2) mild contraction occurring upon fast stretch, but at no angle sufficient to temporarily arrest passive movement (catch): Y=1 (X_V1_=X_V3_); (3) contraction occurring at fast muscle stretch, with an intensity sufficient to temporarily arrest passive movement (catch) at a specific angle X_V3_, different from X_V1_, followed by release: Y=2 (X_V1_>X_V3_); (4) contraction occurring at fast muscle stretch, sufficient to temporarily arrest passive movement (catch) at a specific angle X_V3_, different from X_V1_, followed by a release that is itself sufficient to elicit a second stretch reflex. As the clinician maintains pressure, depending on the movement speed during the release after the second stretch reflex, a new stretch reflex occurs, and so on, until speed slows down to a point below the velocity threshold, the time at which the situation exhausts. This is the case of fatigable clonus: Y=3 (X_V1_>X_V3_); (5) contraction occurring at fast muscle stretch, sufficient to temporarily arrest passive movement (catch) at a specific angle X_V3_, different from X_V1_, followed by a release that is itself sufficient to elicit a second stretch reflex. Depending on the movement speed during the release after the second stretch reflex as the clinician maintains pressure, a new stretch reflex occurs, but the speed of release remains constantly greater than the velocity threshold; the situation persists over 10 seconds of maintained stretch. This is the case of infatigable clonus: Y=4 (X_V1_>X_V3_).

The derived ratio (X_V1_−X_V3_)/X_V1_ represents the coefficient of spasticity, which quantifies spasticity as a proportion of the maximal clinical extensibility of the muscle.

### Step-4: angle of match between maximal active agonist effort and passive and active antagonist resistances

After the visual, nongoniometric assessments of passive movements above, the clinician takes the goniometer and asks the patient to attempt the same movement actively against the resistance of the muscle group evaluated, as far as possible, until the active torque produced by the agonist is matched by the combination of passive resistance and spastic cocontraction from the stretched antagonist. The angle of match, or maximal active range of motion against the antagonist (X_A_), is thus obtained; this parameter *mostly* reflects the impairment of motor command, particularly spastic cocontraction of the antagonist tested as it undergoes major change after lidocaine blocks or repeated botulinum toxin injections into the antagonist.

The ratio (X_V1_–X_A_)/X_V1_ is defined as the coefficient of weakness, which measures the overall impairment of active command against the tested antagonist with respect to its maximal clinical extensibility.

### Step-5: residual angle of match after 15 seconds of maximal amplitude alternating movements

The patient performs the same active movement over the maximal range as measured above, then returns to the starting position and repeats these maximal efforts at fast speed in a fixed amount of time of 15 seconds. The patient is asked to produce repeated maximal efforts (ie, maximal amplitude) at each trial during the 15 seconds and to perform these repetitions at fast speed (X_A15_). It is likely that any amplitude decrement from X_A_ to X_A15_ over that short time mostly reflects central fatigability of motor command.

The ratio (X_A_–X_A15_)/X_A_ is thus defined as the coefficient of fatigability, which quantifies amplitude decrement over a 15-second series, regardless of the maximal amplitude reached over a single movement.

## Appendix B Supplementary materials

Appendix 2 Five Step Assessment of spastic paresis in the upper limb Abbreviations: AD, anterior deltoid; B, brachialis; BB, biceps brachialis; BR, brachio-radialis; CB, coracobrachialis; DIO, dorsal interossei; ECRB, extensor carpi radialis brevis; ECRL, extensor carpi radialis longus; ECU, extensor carpi ulnaris; EDC, extensor digitorum communis; FCR, flexor carpi radialis; FCU, flexor carpi ulnaris; FDP, flexor digitorum profundis; FDS, flexor digitorum superficialis; FPB, flexor pollicis brevis; FPL, flexor pollicis longus, IS, infraspinatus; LD, latissimus dorsi; LHT, long head of triceps; MT, middle trapezius; PI, first phalanx; PII, second phalanx; PIII, third phalanx; PIO, palmar interossei; PM, pectoralis major; PQ, pronator quadratus; PT, pronator teres; Rh, rhomboids; SS, subscapularis; TB, triceps brachialis; Tm, teres minor; TM, teres major; X_A_, angle of match between agonist effort and passive and active resistances from the antagonist; X_A15_, residual angle of match after 15 seconds of repeated maximal amplitude active movements against the resistance of the tested muscle; X_V1_, maximal clinical extensibility; X_V3_, angle of catch or clonus; Y, grade of spasticity. Image, application 1

Appendix 3 Five Step Assessment of spastic paresis in the lower limb Abbreviations: C_F_, coefficient of fatigability; C_SH_, coefficient of shortening; C_SP_, coefficient of spasticity; C_W_, coefficient of weakness; X_A_, angle of match between agonist effort and passive and active resistances from the antagonist; X_A15_, residual angle of match after 15 seconds of maximal amplitude active movements; X_V1_, maximal clinical extensibility; X_V3_, angle of catch or clonus; Y, grade of spasticity. Image, application 2

Appendix 4 Protocol for the Five Step Assessment (FSA) of soleus Abbreviations: X_A_, angle of match between agonist effort and passive and active resistances from the antagonist; X_A15_, residual angle of match after 15 seconds of maximal amplitude active movements; X_V1_, maximal clinical extensibility; X_V3_, angle of catch or clonus; Y, grade of spasticity. Image, application 3

Supplemental material. Mean intra- and inter-rater percent agreement, Fleiss' kappa coefficients ± standard error (SE), [95% CI] and Gwet's agreement coefficients (AC) ± standard error (SE), [IC 0.95%] for Spasticity grade (Y) per muscle group. CI, confidence interval; SE, standard error. Image, application 4

Image, application 5

Image, application 6

Image, application 7

## Footnotes

Supplementary material associated with this article can be found, in the online version, at doi:10.1016/j.arrct.2025.100444.
