# Supplementary material for: Reliability of the Five Step Assessment and Its Coefficients of Impairment in Spastic Paresis
Source: Arch Rehabil Res Clin Transl. 2025 Feb 22;7(2):100444. doi: 10.1016/j.arrct.2025.100444 (PMC12265912; doi:10.1016/j.arrct.2025.100444)
Supplement: Supplementary file 1 — Appendix 2 Five Step Assessment of spastic paresis in the upper limb Abbreviations: AD, anterior deltoid; B, brachialis; BB, biceps brachialis; BR, brachio-radialis; CB, coracobrachialis; DIO, dorsal interossei; ECRB, extensor carpi radialis brevis; ECRL, extensor carpi radialis longus; ECU, extensor carpi ulnaris; EDC, extensor digitorum communis; FCR, flexor carpi radialis; FCU, flexor carpi ulnaris; FDP, flexor digitorum profundis; FDS, flexor digitorum superficialis; FPB, flexor pollicis brevis; FPL, flexor pollicis longus, IS, infraspinatus; LD, latissimus dorsi; LHT, long head of triceps; MT, middle trapezius; PI, first phalanx; PII, second phalanx; PIII, third phalanx; PIO, palmar interossei; PM, pectoralis major; PQ, pronator quadratus; PT, pronator teres; Rh, rhomboids; SS, subscapularis; TB, triceps brachialis; Tm, teres minor; TM, teres major; XA, angle of match between agonist effort and passive and active resistances from the antagonist; XA15, residual angle of match after 15 seconds of repeated maximal amplitude active movements against the resistance of the tested muscle; XV1, maximal clinical extensibility; XV3, angle of catch or clonus; Y, grade of spasticity. [file mmc1.docx]

**Procedures involved in the FIVE-STEP Assessment**

Following and expanding on Tardieu’s insights on *chronic* spastic paresis, and in contrast with the assessment in peripheral paresis (Medical Research Council scale) that assesses *agonist* muscles for their capacity to generate movement against gravity, the four technical steps (Steps 2 to 5) of the Five-Step Assessment evaluate each muscle group for its capacity to *oppose* movement, not to generate it. Steps 2 and 3 rate capacity to oppose *passive* movements at two different velocities, while Steps 4 and 5 rate the capacity to oppose active attempts at movement, before and after fatigue.

Each step of the assessment yields a quantitative continuous variable, making it possible to monitor status. For the technical steps, the angles determined are not based on joint positions, but based on zero being the theoretical angle of minimal stretch of the muscle group assessed. This point of minimal stretch of the muscle group is taken as the point of reference where resistance starts. Steps 2 and 3 constitute what has been known in the literature as the Tardieu Scale. These two angles have yielded good to excellent intra- and inter-rater reliability, both in children and adults, without the use of a goniometer.

***Step-2: Angle of arrest at slow speed of stretch (X_V1_)***

Each muscle group is first evaluated using very slow and powerful stretch, without jeopardizing soft tissue integrity or patient tolerance. The angle at which muscle resistance is no longer overcome by the examiner with respect to soft tissue integrity and patient comfort is defined as the maximal passive clinical extensibility of the muscle group assessed. X_V1_ *mostly* reflects muscle shortening (as opposed to joint adherences), more so than muscle overactivity (spastic dystonia) as it undergoes little change after lidocaine blocks or repeated botulinum toxin injections.

X_V1_ is then appreciated with respect to the expected normal passive amplitude, X_N_, defining the ratio (X_N_ – X_V1_)/X_N_ as the coefficient of shortening (C_SH_) of the tested muscle.

***Step-3: Angle of Catch or Clonus (X_V3_) and Spasticity Grade (Y) – The Tardieu Scale***

Each muscle group is then evaluated using fast stretch, i.e., stretch at the fastest possible speed for the examiner (V3, fast velocity), *without forcing the way beyond the muscle catch*. According to the Tardieu Scale, the clinician derives two parameters from this maneuver. The angle of catch or clonus (X_V3_) represents the threshold to elicit the reflex. The *spasticity grade* Y, i.e. the type of muscle reaction that occurs upon fast stretch at that angle, clinically reflects the *gain* of the stretch reflex. Five situations may occur as follows: 1) no muscle contraction upon fast stretch: Y=0 (X_V1_ = X_V3_); 2) mild contraction occurring upon fast stretch, but at no angle sufficient to temporarily arrest passive movement (catch): Y=1 (X_V1_ = X_V3_); 3) contraction occurring at fast muscle stretch, with an intensity sufficient to temporarily arrest passive movement (catch) at a specific angle X_V3_, different from X_V1_, followed by release: Y=2 (X_V1_ > X_V3_); 4) contraction occurring at fast muscle stretch, sufficient to temporarily arrest passive movement (catch) at a specific angle X_V3_, different from X_V1_, followed by a release that is itself sufficient to elicit a second stretch reflex. As the clinician maintains pressure, depending on the movement speed during the release after the second stretch reflex a new stretch reflex occurs, and so on until speed slows down to a point below the velocity threshold, time at which the situation exhausts. This is the case of fatigable clonus: Y=3 (X_V1_ > X_V3_); 5) contraction occurring at fast muscle stretch, sufficient to temporarily arrest passive movement (catch) at a specific angle X_V3_, different from X_V1_, followed by a release that is itself sufficient to elicit a second stretch reflex. Depending on the movement speed during the release after the second stretch reflex as the clinician maintains pressure, a new stretch reflex occurs, but speed of release remains constantly greater than the velocity threshold; the situation persists over 10 seconds of maintained stretch. This is the case of unfatigable clonus: Y=4 (X_V1_ > X_V3_).

The derived ratio (X_V1_ – X_V3_)/X_V1_ represents the coefficient of spasticity (C_SP_), which quantifies spasticity as a proportion of the maximal clinical extensibility of the muscle.

***Step-4: Angle of match (X_A_) between maximal active agonist effort and passive and active antagonist resistances***

After the visual, non-goniometric assessments of passive movements above, the clinician takes the goniometer and asks the patient to attempt the same movement actively against the resistance of the muscle group evaluated, as far as possible, until the active torque produced by the agonist is matched by the combination of passive resistance and spastic co-contraction from the stretched antagonist. The Angle of Match, or maximal active range of motion against the antagonist*,* X_A_ is thus obtained; this parameter *mostly* reflects the impairment of motor command, particularly spastic cocontraction of the antagonist tested as it undergoes major change after lidocaine blocks or repeated botulinum toxin injections into the antagonist.

The ratio (X_V1_ – X_A_)/X_V1_ is defined as the coefficient of weakness (C_W_), which measures the overall impairment of active command against the tested antagonist, with respect to its maximal clinical extensibility.

***Step-5: Residual Angle of Match after 15 seconds of Maximal Amplitude Alternating Movements (X_A15_)***

The patient performs the same active movement over the maximal range as measured above, then returns to the starting position and repeats these maximal efforts at fast speed in a fixed amount of time of 15 seconds. The patient is asked to produce repeated maximal efforts (*i.e*. of maximal amplitude) at each trial during the 15 seconds, and to perform these repetitions at fast speed. It is likely that any amplitude decrement from X_A_ to X_A15_ over that short time mostly reflects central fatigability of motor command.

The ratio (X_A_ – X_A15_)/X_A_ is thus defined as the coefficient of fatigability (C_F_), which quantifies amplitude decrement over a 15-second series, regardless of the maximal amplitude reached over a single movement.
