# Supplementary material for: Reliability of the Five Step Assessment and Its Coefficients of Impairment in Spastic Paresis
Source: Arch Rehabil Res Clin Transl. 2025 Feb 22;7(2):100444. doi: 10.1016/j.arrct.2025.100444 (PMC12265912; doi:10.1016/j.arrct.2025.100444)
Supplement: Supplementary file 7 [file mmc7.docx]

**SUPPLEMENTAL MATERIAL LEGEND**

**Supplemental material.** Mean intra- and inter-rater percent agreement, Fleiss' kappa coefficients ± standard error (SE), [95% CI] and Gwet’s agreement coefficients (AC) ± standard error (SE), [IC 0.95%] for Spasticity grade (Y) per muscle group.

CI, confidence interval; SE, standard error.

**APPENDIX LEGENDS**

**Appendix 1.** Procedures Involved in the Five-Step Assessment.

**Appendix 2.** Five Step Assessment of spastic paresis in the upper limb.

X_V1_, maximal clinical extensibility; X_V3_, angle of catch or clonus; Y, grade of spasticity; X_A_, angle of match between agonist effort and passive and active resistances from the antagonist; X_A15_, residual angle of match after 15 seconds of repeated maximal amplitude active movements against the resistance of the tested muscle; AD, anterior deltoid; AP, adductor pollicis; BB, biceps brachialis; B, brachialis; BR, brachio-radialis; CB, coracobrachialis; ECU, extensor carpi ulnaris; EDC, extensor digitorum communis; ECR, extensor carpi radialis; FCR, flexor carpi radialis; FCU, flexor carpi ulnaris; FDS, flexor digitorum superficialis; FDP, flexor digitorum profundis; FPL, flexor pollicis longus, FPB, flexor pollicis brevis; IOD, interossei dorsales, IOP, interossei palmares; IS, infraspinatus; LD, latissimus dorsi; LHT, long head of triceps; MT; PM, pectoralis major; PQ, pronator quadratus; PT, pronator teres; PL, palmaris longus; PI, first phalanx; PII, second phalanx; PIII, third phalanx; TM, teres major; Rh, rhomboids; SS, subscapularis; Tm, teres minor; TB, triceps brachialis.

**Appendix 3**. Five Step Assessment of spastic paresis in the lower limb.

*Abbreviations:* X_V1_, maximal clinical extensibility; X_V3_, angle of catch or clonus; Y, grade of spasticity; X_A_, angle of match between agonist effort and passive and active resistances from the antagonist; X_A15_, residual angle of match after 15 seconds of maximal amplitude active movements.

**Appendix 4:** Protocol for the Five Step Assessment of soleus
