# Supplementary material for: Reliability of the Five Step Assessment and Its Coefficients of Impairment in Spastic Paresis
Source: Arch Rehabil Res Clin Transl. 2025 Feb 22;7(2):100444. doi: 10.1016/j.arrct.2025.100444 (PMC12265912; doi:10.1016/j.arrct.2025.100444)
Supplement: Supplementary file 7 [file mmc5.docx]

	Intra-rater					Inter-rater				
	Percent Agreement	Fleiss' Kappa		Gwet's AC		Percent Agreement	Fleiss' Kappa		Gwet's AC	
	Coefficient ± SE	Coefficient ± SE	95% CI	Coefficient ± SE	95% CI	Coefficient ± SE	Coefficient ± SE	95% CI	Coefficient ± SE	95% CI
Shoulder extensors	0.99±0.01	0.54±0.11	[0.33-0.76]	0.99±0.01	[0.98-0.99]	0.99±0.01	0.58±0.15	[0.28-0.89]	0.99±0.01	[0.98-1.00]
Elbow flexors	0.99±0.01	0.70±0.07	[0.55-0.84]	0.99±0.01	[0.98-1.00]	0.99±0.01	0.70±0.11	[0.47-0.93]	0.99±0.01	[0.98-1.00]
Wrist flexors	0.98±0.01	0.54±0.06	[0.41-0.66]	0.97±0.01	[0.96-0.98]	0.98±0.01	0.46±0.11	[0.23-0.69]	0.98±0.01	[0.96-0.99]
Finger flexors	0.98±0.01	0.64±0.06	[0.51-0.77]	0.97±0.01	[0.96-0.98]	0.98±0.01	0.61±0.10	[0.39-0.83]	0.97±0.01	[0.96-0.98]
Gluteus maximus	0.98±0.01	0.31±0.10	[0.10-0.52]	0.98±0.01	[0.97-0.99]	0.99±0.01	0.36±0.15	[0.03-0.68]	0.98±0.01	[0.97-1.00]
Rectus femoris	0.98±0.01	0.64±0.03	[0.56-0.70]	0.95±0.01	[0.94-0.96]	0.98±0.01	0.61±0.06	[0.49-0.73]	0.96±0.01	[0.94-0.98]
Soleus	0.99±0.01	0.87±0.06	[0.74-0.99]	0.99±0.01	[0.98-0.99]	0.99±0.01	0.84±0.12	[0.59-1.00]	0.98±0.01	[0.97-1.00]
Gastrocnemius	0.99±0.01	0.62±0.08	[0.44-0.79]	0.98±0.01	[0.96-1.00]	0.98±0.01	0.59±0.13	[0.31-0.86]	0.98±0.01	[0.96-1.00]
